# An Investigation of a Natural Biosorbent for Removing Methylene Blue Dye from Aqueous Solution

**DOI:** 10.3390/molecules28062785

**Published:** 2023-03-20

**Authors:** Basma G. Alhogbi, Ghadeer S. Al Balawi

**Affiliations:** Department of Chemistry, Faculty of Science, King Abdulaziz University, Jeddah 21589, Saudi Arabia

**Keywords:** environmentally friendly product, fiber of palm tree, kaolin, zeolite, dye retention, adsorption

## Abstract

T he current study reports the use of zeolite prepared from a kaolin composite via physical mixing with different ratios from fiber of palm tree (Zeo-FPT) as a sustainable solid sorbent for the removal of methylene blue (MB) dye from aqueous solutions. The prepared biosorbent was fully characterized using XRD, TGA, SEM, and FTIR. The impacts of various analytical parameters, for example, contact time, dosage, MB dye concentration, and the pH of the solution, on the dye adsorption process were determined. After a contact time of 40 min, the capacity to remove MB dye was 0.438 mg g^−1^ at a Zeo-FPT composition ratio of 1F:1Z. At pH 8, Zeo-FPT (1F:1Z) had a removal efficiency of 87% at a sorbent dosage of 0.5 g for a concentration of MB dye in an aqueous phase of 10 mg L^−1^. The experimental data were also analyzed using the kinetic and adsorption isotherm models. The retention process fitted well with the pseudo-second-order model (R^2^ 0.998), where the *Q_e,calc_* of 0.353 mg g^−1^ was in acceptable agreement with the *Q_e,exp_* of 0.438 mg g^−1^. The data also fitted well with the Freundlich isotherm model, as indicated by the correlation coefficient value (R^2^ 0.969). The Zeo-FPT attained a high percentage (99%) in the removal of MB dye from environmental water samples (tap water, bottled water, and well water). Thus, it can be concluded that the proposed zeolite composite with fiber of palm tree (Zeo-FPT) is a suitable, environmentally friendly, and low-cost adsorbent for removing dyes from wastewater.

## 1. Introduction

Recently, from a global perspective, environmental protection awareness has increased. Following the industrial revolution and the rapid development of new technologies, a great deal of waste has been produced that is harmful to the environment and to public health. Water bodies are most affected, resulting in the water becoming unsuitable for consumption. Dyes are used in many industries, such as textiles, food, and cosmetics, and are also used for medical purposes, including as antiseptic agents, making them common water pollutants [1]. During the dyeing process, many chemicals are discharged into the water, affecting living organisms, damaging the soil and plants, and poisoning drinking water. For example, the dye methylene blue (MB) is a heterocyclic aromatic compound, referred to as 3, 7 bis (dimethyl amino) phenothiazine-5-ium chloride by the International Union of Pure and Applied Chemistry (IUPAC). It has been defined as a biologically active substance and is a cationic thiazine dye that, when oxidized, has a deep blue color, but when reduced to leucomethylene blue, it becomes colorless [2,3]. At different doses, MB dye is toxic to animals, aquatic ecosystems, and humans; for example, at high doses, it may cause chest pain, dyspnea, restlessness, tremors, and damage to the digestive and respiratory systems of humans [4,5].

Recently, much attention has been directed toward developing low-cost and widely applicable technologies to meet the significant challenge of the treatment of wastewater contaminated with dyes. The elements of the dyes vary with respect to their chromosphere groups and substituents, thus affecting characteristics such as polarity and solubility. The most widely used methods for removing dyes from wastewater include coagulation [6], ozonation [7], chlorination [8], electrochemical processes [9], chemical precipitation [10], membrane methods [11], biological treatments [12] and adsorption [13]. The removal of MB dye from wastewater is extremely important, due to the serious environmental damage that it can cause [14,15]. The most common method of dye removal is adsorption as there are a large number of adsorbents available in the field. Adsorption is considered a surface phenomenon with several mechanisms for the inorganic and organic removal of pollutants. Adsorption is a mass transfer operation by which a substance is conveyed from a liquid stage onto a solid surface and becomes bound by chemical and/or physical interactions. The massive surface area causes high surface reactivity and adsorption capacity [16]. Adsorption procedures are preferred because of their advantages in terms of low cost, reliability, ease of process, and, most crucially, the possibility of acquiring the adsorbent from a wide range of natural, artificial, and waste materials, such as lignocellulosic biomass [16].

Presently, the search for low-cost and effective solid-phase extractors that are applicable for the removal of dye from wastewater is ongoing [12]. The efficiency of this process can also be successfully increased by optimizing the conditions through the use of various surface modifications [12]. In the literature, hydroxyapatite modified by magnetic particles is utilized as an effective, stable, biodegradable adsorbent that more easily and rapidly performs the process of removing heavy metal ions, inorganic elements, and organic dyes that constitute environmental pollution [10]. Materials classified as natural adsorbents include clays, charcoal, zeolites, ores, and biomass [13,16]. These natural elements are generally inexpensive and present considerable potential for alteration, with the aim of eventually improving their adsorption abilities [14]. Every adsorbent has its own features in terms of pore structure, porosity, and the essence of its adsorbing surfaces. Some of the waste elements used include coconut shell, fruit waste, bark, scrap tires, rice husk, sawdust, fertilizer waste, petroleum waste, sugar industry waste, palm fiber, fly ash, seafood processing waste, chitosan, peat moss, red mud, clays, and zeolites [1,13,17].

Adsorption technology includes an effective range of techniques for removing most color pigments from wastewater using modified synthesized zeolite to enhance the adsorption capacity via cation exchange [18]. The adsorption of organic dyes using clay has been shown to be a cost-effective approach for the purification of wastewater [13,16]. Many researchers have studied synthetic zeolite, which is promising and relatively inexpensive, and is an ideal scavenger of water pollutants such as organic dyes and heavy metals by means of adsorption and ion exchange. Moreover, zeolite is already used in several applications due to its surface area and high ion exchange capacity [19,20,21]. Zeolites are microporous, crystalline hydrated aluminosilicates with symmetrically stacked A1O_4_ and SiO_4_ tetrahedral forms, which result in an open and stable three-dimensional honeycomb structure with a negative charge within the pores that can be neutralized by positively charged ions (cations) such as sodium. The structural and chemical formula of the crystallographic unit cell zeolite with oxides is Mx/n [(SiO_2_)y (A1O_2_)x] m H_2_O. In this formula, M is exchangeable metal cations, n is the valence of the cation (M), x and y are the total number of tetrahedra per unit cell, and m is the number of water molecules per unit cell [22]. Zeolites are distinguished by the mobile actions inside their channels and cages [23].

Currently, date palm trees are generally found in tropical and subtropical regions of Saudi Arabia and Asia, and they have been essential for the daily routine of citizens inhabiting the Arabian Peninsula for the last 7000 years [24]. A normal date palm tree produces 35 kg of fiber of palm tree (FPT) annually, which is a burnable waste that consists of dried leaves, spathes, sheaths and petioles [25]. This waste is burned on ranches, causing ecological contamination and the demise of important soil microorganisms [26]. Due to their highly lignocellulosic composition and low ash content, date palm tree components have been intensively assessed recently with respect to their suitability for assembling enacted carbon at minimal expense [25]. In contrast to commercial activated carbon, the activated carbon obtained from date palm trees has prominent textural qualities that provide it with a notably greater adsorption limit for water remediation purposes [27]. The important biomass components of fiber of palm tree (FPT) include hemicellulose (18%), cellulose (46%), ash (10.54%), and lignin (20%) [28]. Moreover, lignocellulosic fibers are renewable, biodegradable, recyclable, and supportable materials [29]. Therefore, fiber of palm tree (FPT) can be used as an adsorbent material for wastewater management. In addition, it has been shown that palm fiber can be altered to fully activate carbon for MB dye removal [14,30].

Adsorption processes using zeolite and biosorbents such as fiber of palm tree (FPT) are becoming the most typical and low-cost solution for removing organic pollutants from wastewater such as effluent bodies polluted by industry [15,23,31]. Thus, this study is focused on (i) testing the efficiency of zeolite prepared from local Saudi kaolin and composite with fiber of palm tree (FPT) physical mixing as a sustainable sorbent for obtaining a more cost-effective material for use as an adsorbent in wastewater treatment for MB dye; (ii) Biosorbent characterization of the prepared biosorbent and finally; (iii) calculating the adsorption efficiencies of various zeolite–fiber ratios as a function of contact time, adsorption pH, sorbent dosages, and different concentrations. The data that can be obtained from the kinetic and isotherm models will help to establish the most probable retention mechanism of MB dye by Zeo-FPT sorbent in the test aqueous solution. Moreover, the trace spectrophotometric determination of MB dye in a variety of environmental waters was attempted.

## 2. Results and Discussion

### 2.1. Adsorbent Characterization

**Figure 1:**
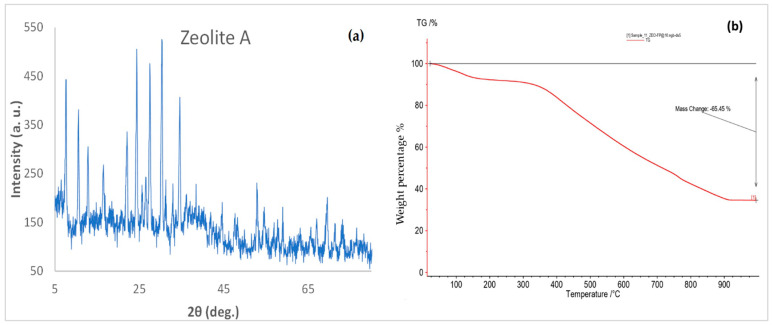
XRD diffractogram of zeolite A (**a**) and TGA thermographs analysis of Zeo-FPT composite (**b**).

The prepared biosorbent sample with mixed physical composite (Zeo-FPT) was fully characterized using different techniques, i.e., XRD, TGA, SEM, and FTIR. The lattice constant of synthesized zeolite A is illustrated in Table 1. The XRD pattern of the percentage crystal of the zeolite A structure was used to calculate the intensities of the peaks at 2θ values of 7.18, 10.17, 12.46, 21.68, 24.06, 27.14, 29.97, 34.21, 52.89, and 54.43, which are presented in Figure 1a. The Scherrer formula was successfully used to calculate the crystallite size of the zeolite A, which was found to be 254 Å (25.4 nm) [20,22].

Thermogravimetric (TGA) analysis was used to evaluate the mass sample transformation and thermal stability at different temperatures. The TGA of the Zeo-FPT showed three phases, as presented in Figure 1b. The first phase appeared at an approximate range of 100–150 °C, accompanied with a loss of weight of 8.9% due to dehydration of water molecules. The second degradation phase happened around 200–300 °C, which may have been due to the dehydroxylation of the Zeo-FPT [32]. The final phase occurred at a wider range of 300–600 °C and can be attributed to decomposition of the carbonic natural material in the fiber (lignin) [33]. The percentage decomposition of mass loss was 65.45% at the end of all thermal phases. This indicates that Zeo-FPT showed an appropriate thermal stability up to high temperatures.

**Figure 2:**
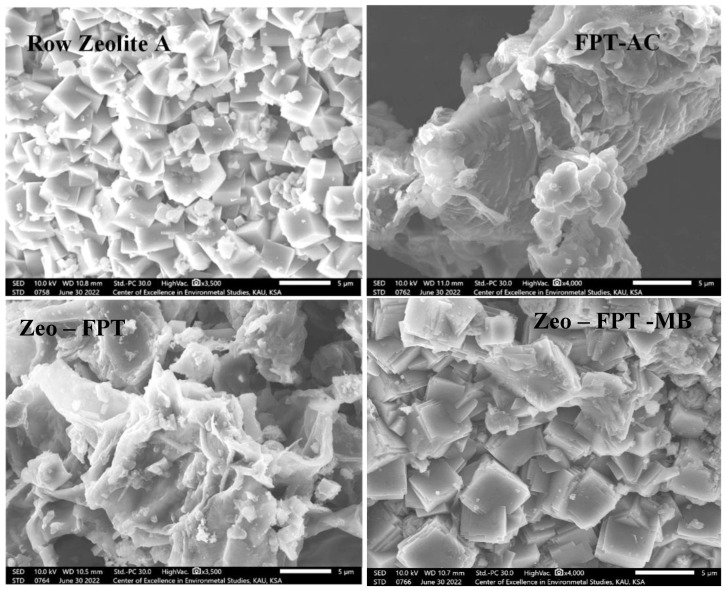
**The** SEM images of synthesized pure zeolite A, FPT-AC, Zeo-FPT composite before sorption, and Zeo-FPT-MB after sorption.

The SEM images of synthesized pure zeolite A (Zeo.), FPT-AC, Zeo-FPT, and Zeo-FPT-MB are shown in Figure 2. These images reveal that the surface of zeolite A was characterized by a rhombohedral crystal (pseudocubic) morphology. FPT-AC and Zeo-FPT presented irregular aggregates, rough surfaces, porous cavities on the surface and different particle sizes. There was partial darkness in the blue color after the MB dye was adsorbed on the pseudocubic crystals of the Zeo-FPT-MB, indicating the irregular and abundant adsorption of dye on the sorbent surface [18].

**Figure 3:**
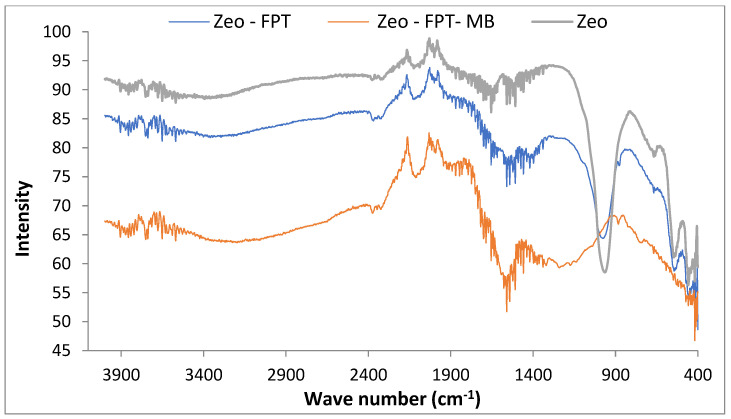
FTIR spectra of Zeo, the Zeo-FPT composite, and Zeo-FPT-MB dye after adsorption.

The FTIR spectroscopy results shown in Figure 3 and Table 2 were used to examine the chemical structures of the Zeo-FPT absorbent before and after the removal of the MB dye. In contrast to the spectrum of Zeo, Zeo-FPT exhibited an absorption band intensity that decreased at 902 cm^−1^, which may be proof of the validity of the combination of zeolite and FPT. The adsorption of the MB dye onto Zeo-FPT showed that the spectrum was controlled by the substitution of a broad band around 1008–902 cm^−1^, probably due to the MB dye interacting with the functional groups of Si–O–T (T = Si or Al) present on the Zeo-FPT surface [34,35].

### 2.2. Optimization of the Dye Retention by the Established Extractor

**Figure 4:**
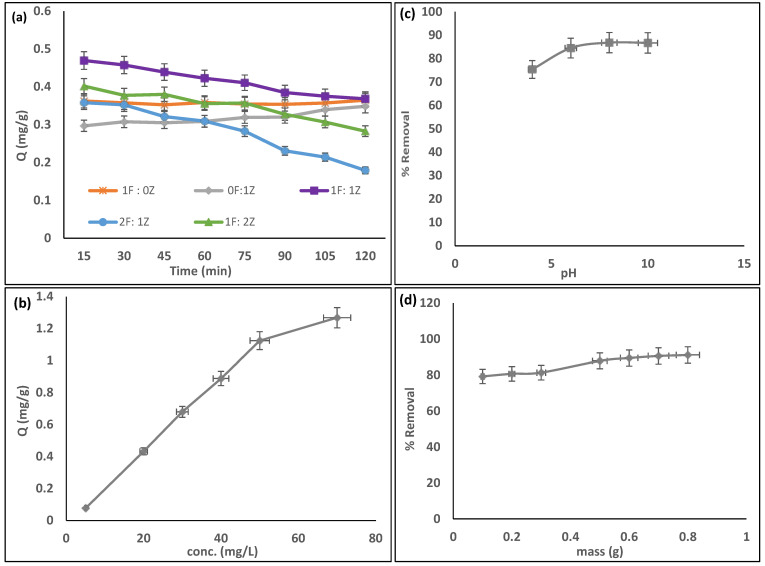
The effects of different factors on the MB dye removal capacity of Zeo-FPT: (**a**) contact time at different ratios of Zeo-FPT; (**b**) solution concentration of Zeo-FPT (1F:1Z); (**c**) the removal percentage at different pH values; and (**d**) the mass of Zeo-FPT (1F:1Z).

The effects of different parameters on Zeo-FPT adsorption efficiency were investigated. The effects of different contact times of 0.5 g of different ratios of fiber (F), zeolite (Z), and the composite (Zeo-FPT) (0F:1Z, 1F:0Z, 1F:1Z, 1F:2Z, and 2F;1Z) with 25 mL of 10 mg L^−1^ MB dye solution were combined with different shaking durations (15, 30, 45, 60, 75, 90, 105, and 120 min) at 300 rpm at room temperature. The results shown in Figure 4a indicate that in the first 15 to 90 min, the removal capacity (Q) of 0F:1Z for the MB dye was around 0.291 mg g^−1^, while that of 1F:0Z was 0.356 mg g^−1^ at 120 min, and the removal capacity increased to 0.348 mg g^−1^ for 0F:1Z and 0.368 mg g^−1^ for 1F:0Z. Therefore, with increasing contact time, the removal capacity increased, and adsorption reached equilibrium. This indicates that there were free adsorbent sites and an intense interaction between the MB dye and the adsorbent sites involved. The results shown in Figure 4a for 1F:2Z indicate that there was a gradual decline in the adsorption capacity (Q), which began at 0.4 mg g^−1^ to 0.3 mg g^−1^ at 120 min, after which there was an increase, and the final Q value was 0.402 mg g^−1^. The results for 2F:1Z show that there was a gradual decline in the adsorption capacity (Q), which began around 0.35 mg g^−1^ to 0.2 mg g^−1^ at 120 min, after which there was an increase, and the final Q value was 0.358 mg g^−1^. Moreover, the results for 1F:1Z show that there was a gradual decline in the adsorption capacity (Q), which began at 0.47 mg g^−1^ to 0.36 mg g^−1^ at 120 min. Hence, an increase in the contact time resulted no significant change in the removal capacity, which means there was sufficient time for the adsorbate to adsorb into the layer of the adsorbate. The highest MB dye removal capacity was displayed by 1F:1Z—0.438 mg g^−1^ at 40 min. Dosa et al. (2022) [20] investigated the use of Clinoptilolite, a natural zeolite, in the adsorption of MB dye, and found that the efficiency of the removal of the MB dye was nearly 96% at 210 min. Thus, the ratio of 1F:1Z of Zeo-FPT was selected to carry out further investigations of the different factors, such as the pH of the solution, the MB concentration, and the adsorbent dosage.

The variations in the efficiency of Zeo-FPT in removing MB dye were tested at different pH levels. The solution was composed of 0.2 g 1F:1Z and 25 mL of 50 mg L^−1^ MB dye, and was introduced at pH values of 4, 6, 8, and 10 for 40 min. The results regarding the pH effect are presented in Figure 4b. The lowest percentage removal was 75.35% at pH 4, and in a slightly acidic medium, at pH 6, there was a minor increase to 84.48%. Finally, at pH 8 and 10, the activity remained stationary at 86.8%, which represents the highest removal percentage. The lower adsorption of MB dye in acidic pH solution was probably due to the fact that, in an acidic pH suspension, the surface may become positively charged, thus causing H^+^ ions to effectively compete with MB dye cations for the active sites of Zeo-FPT. This may cause a decrease in the amount of dye adsorbed [36]. However, the cationic MB dye at different ranges of pH showed a high percentage removal 84.48%, which makes the Zeo-FPT a significant sorbent for wastewater remediation. A similar result was found when removing MB dye from textile wastewater using zeolite-x and kaolin adsorbents; the removal percentages were 97.77% at pH 4 and 86.86% at pH 6, respectively [6].

The effect of various concentrations of MB dye solution obtained by mixing 0.2 g of 1F:1Z from Zeo–FPT with 25 mL of (5, 20, 30, 40, 50 or 70 mg L^−1^) for 40 min is shown in Figure 4c. There was a rapid rise in the adsorption capacity (Q) from 0.07 to 1.12 mg g^−1^ for the 5 mg L^−1^ to 50 mg L^−1^ MB dye concentration. The maximum adsorption capacity (Q) was 1.26 mg g^−1^, which was attained by the 70 mg L^−1^ MB dye concentration; this was due to the increase in the driving force of the concentration gradient as a result of the increase in the initial dye concentration. Li et al. (2015) [37] found a similar pattern when investigating the ability of zeolite synthesized from electrolytic manganese to remove MB dye from wastewater, showing that the amount of adsorbed MB increased with an increasing MB dye concentration.

Further evaluations of the effect of Zeo-FPT (1F:1Z) on the MB dye adsorption process were carried out. The relationship between the sorbent dosages and the percentage removal was investigated. Figure 4d presents the different masses of Zeo-FPT (0.1, 0.2, 0.3, 0.5, 0.6, 0.7, and 0.8 g) in 25 mL of 10 mg L^−1^ MB dye solution after 40 min at room temperature. It was observed that the percentage removal at 0.1 g was 79.3%, followed by a gradual increase at 0.3 g to 81.4%, and another increase at 0.8 g to stability at 91.2%. The percentage removal demonstrates an increasing trend with increasing adsorbent dosages. This is because, at higher Zeo-FPT masses, there was a greater availability of active sites, and they were surrounded by higher amounts of MB dye-adsorbent molecules [36].

### 2.3. Sorption Kinetics

**Figure 5:**
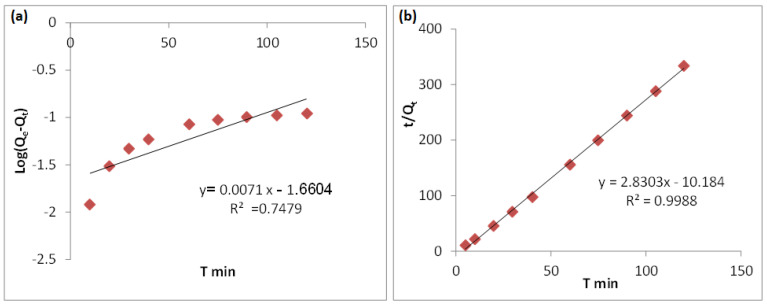
Adsorption kinetics: (**a**) pseudo-first-order model and (**b**) pseudo-second-order model for the removal of MB using Zeo-FPT.

The relationship between the concentration of Zeo-FPT (1F:1Z) and adsorption time was determined using pseudo-first- and pseudo-second-order kinetics. The results in Figure 5a,b show that the correlation coefficient R^2^ was 0.748 in the pseudo-first-order kinetics compared with R^2^ 0.998 in the pseudo-second-order kinetics. The values of the adsorption kinetics parameters in Table 3 show that the sorption capacity of the pseudo-second-order model was *Q_e,calc_* 0.353 mg g^−1^ for the Zeo-FPT at equilibrium, which was estimated to be similar to the experimental data of *Q_e,exp_* 0.438 mg g^−1^. The constant rate of adsorption K_2_ was −0.787 (g mg^−1^ min^−1^). This indicates that the adsorption rate-limiting step followed the pseudo-second-order kinetics model, and the adsorption rate of Zeo-FPT was dependent on the MB dye concentration. The capability of the synthesized zeolite 4A adsorbent from Ethiopian kaolin was investigated regarding the removal of MB dye. The data fit best to pseudo-second-order kinetics [13]. Alhogbi et al. (2021) [30] studied activated carbon (AC) from palm tree fiber (PTF) waste for the removal of Congo red (CR), an anionic dye, and Rhodamine B (RhB), a cationic dye, from wastewater; the results show that the adsorption mechanism followed a pseudo-second-order model. Huang et al. (2019) [38] studied the removal efficiency of MB dye onto modified zeolite via magnetic graphene oxide (Cu-Z-GO-M) composites using two ratios (1:2 and 1:1), and found that the adsorption behavior followed pseudo-second-order kinetics.

### 2.4. Adsorption Isotherm

**Figure 6:**
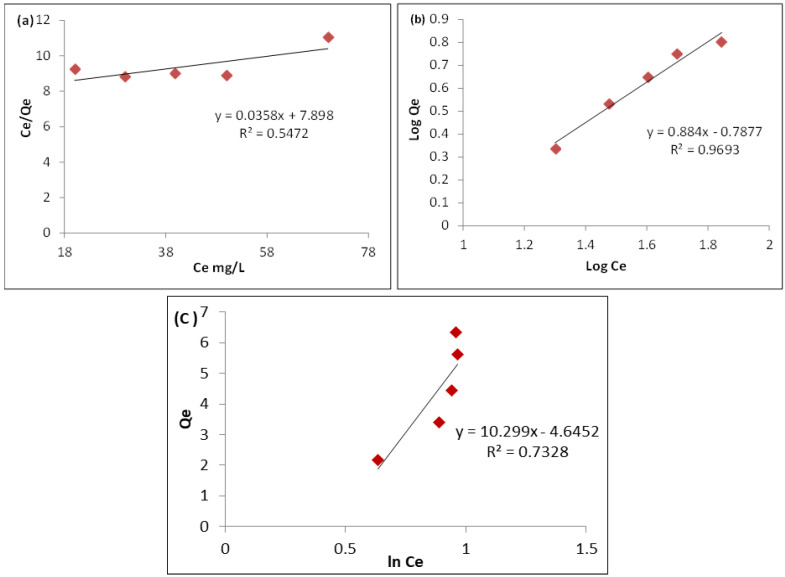
Adsorption isotherms (Langmuir model (**a**) Freundlich model (**b**) and Temkin model (**c**)) for removal of MB dye using Zeo-FPT.

The adsorption isotherm shows the correlation between the adsorbate adsorbed by the adsorbent (*Q_e_*) and that remaining in solution after reaching equilibrium (*C_e_*). The adsorption isotherm models are shown in Figure 6a–c; the value of the correlation coefficient of R^2^ in the Langmuir model was 0.547. The adsorption mechanism followed the Freundlich model with a correlation coefficient of R^2^ 0.969 (linear); this indicates that the MB dye was adsorbed in multiple layers and was heterogeneous on the surface of Zeo-FPT (1F:1Z). Based on the results presented in Table 4, *1/n* = 0.88, which indicates that the sorption was in favorable [38]. The data applied to the Temkin isotherm model indicate that the adsorption of MB dye onto Zeo-FPT (1F:1Z) was inferior, with the R^2^ of 0.732 and the adsorption heat of 9.36 kJ mol^−1^. Domingues et al. (2020) [27] studied the use of *Zygia cauliflora* seeds for water purification, and found that the adsorption mechanism was best described by the Freundlich isotherm model.

Table 5 contains a list of results from recent research conducted on the removal of MB dye from wastewater using natural solid sorbents. Each study had a different method of eliminating MB dye from the aqueous solution. However, in this study, the removal capacity of MB dye onto Zeo-FPT (*Q_e,exp_* 0.438 mg g^−1^) at 25 °C within 40 min proved that Zeo-FPT (1F:1Z) has potential for use in the removal of MB dye from an aqueous solution with minimal time and an adequate removal capacity.

### 2.5. Environmental Samples

Different environmental samples such as tap water, well water, and bottled water were collected to study the efficiency of the Zeo-FPT (1F:1Z) composite. Environmental sampling was performed to establish whether different sources of water could be purified to the maximum extent through adsorption by Zeo-FPT. The results are shown in Table 6. The removal percentages of tap water, well water, and bottled water were 99.04%, 99.38%, and 97.86%, respectively. The adsorption capacities (*Q*) of tap water, well water, and bottled water were 1.238 mg g^−1^, 1.242 mg g^−1^, and 1.22 mg g^−1^, respectively. The obtained results are outstanding; they show a high removal percentage and removal capacity of MB dye from tap water, bottled water, and well water. This confirms that Zeo-FPT has high potential utility as a solid adsorbent for the removal of MB dye from environmental water samples.

## 3. Experimental

### 3.1. Chemicals and Materials

The two main raw materials used in this investigation, i.e., kaolin clay and fiber of palm tree (FPT), were supplied from the northern part of Saudi Arabia by the Saudi Geological Survey, and the FPT was collected from Jeddah city, in the Western Region of Saudi Arabia. The MB dye chosen as the target compound has the molecular formula C_16_H_18_ClN_3_S and a molecular weight of 319.09 g mol^−1^. A high purity methylene blue dye (Sigma–Aldrich, Darmstadt, Germany) was used, and sodium hydroxide (NaOH, Riedel-deHaen Laboratories Supplies, Wycombe, UK) was used to control the pH.

### 3.2. Instrumentation

The synthesized samples of zeolite (Z), fiber of palm tree active carbon (FPT-AC) and composite (Zeo-FPT) before and after adsorption of MB dye were evaluated and characterized based on their morphology and phase analysis. The crystalline stage and crystallite size of the zeolite (Z) sample were examined by X-ray powder diffraction (XRD) on a Rigaku XRD system with a RINT 2000 broad-angle goniometer with a power of 40 kV × 30 mA and Cu Kα radiation (λ = 0.15478 nm). The 2Ɵ intensity information was determined at temperatures ranging from 10 to 80 °C. The thermal stability of the developed materials was analyzed using the thermogravimetric analyzer (TGA) under a N_2_ atmosphere at 10–1000 °C. A scanning electron microscope (SEM) is an essential apparatus for characterizing mesoporous and microporous molecular sieve elements. The obtained micrographs show the particle morphology of the synthesized materials and the presence of any amorphous stages in the selected samples [16]. The samples were analyzed using a field emission JEOL JSM-7600F SEM. The composition of solids and liquids was revealed by Fourier transform infrared spectroscopy (FTIR). Briefly, FTIR analysis evaluates a sample’s absorption of infrared light at various wavelengths to ascertain the material’s molecular composition and structure by exposing it to infrared (IR) radiation.

### 3.3. Preparation of the Biosorbent

**Scheme 1:**
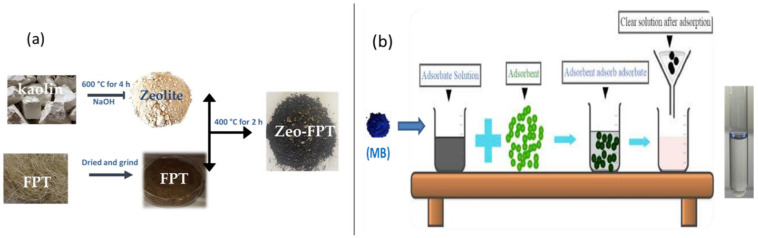
Schematic illustrations of the required steps for the production of the biosorbent Zeo-FPT (**a**) and batch experiment (**b**).

Zeolite A was prepared from Saudi kaolin using the hydrothermal treatment method mentioned in [31,41]. A certain amount of Saudi kaolin was calcinated at 600 °C for 4 h, then left in an oven for 24 h. Calcined kaolin was mixed with NaOH dissolved in distilled water, stirred, and left at room temperature for 24 h. The synthesized zeolite was kept in a water bath for 6 h at 60 °C, after which it was filtered and washed with distilled water until the pH reached 7–8, then left to completely dry [41]. The fiber of palm tree (FPT) was washed several times with distilled water to remove surface impurities and dust, and was left to completely dry at room temperature, then was ground and sifted. The carbonized fiber was placed in a muffle furnace at 400 °C for a duration of 120 min using a porcelain crucible with lid to lower oxygen release, as shown in Scheme 1a.

### 3.4. Recommended Batch Experiments

The batch experiment shown in Scheme 1b was used to investigate the adsorption performance of prepared samples with different ratios of a fiber (F) and zeolite (Z) composite (Zeo-FPT) (0F:1Z, 1F:0Z, 1F:1Z, 1F:2Z and 2F;1Z). The effect of contact time was determined with 25 mL of 10 mg L^−1^ MB dye solution added to 0.5 g of sorbent and shaken for different periods of time (15, 30, 45, 60, 75, 90, 105, and 120 min) at 300 rpm at room temperature. Then, the samples were filtered using No. 2 Whatman filter paper. The filtered samples were analyzed to determine the MB dye concentration using UV–vis spectrophotometry at a maximum absorbance of λ_max_ = 664 nm to study the efficiency of the Zeo-FPT composite as a sorbent material based on different factors, such as the effect of the solution’s pH (4, 6, 8, and 10), different MB dye concentrations (5, 20, 30, 40, 50, 70, and 100 mg L^−1^), and different sorbent dosages (0.1, 0.2, 0.3, 0.5, 0.6, 0.7, and 0.8 g). It is important to note that the homogeneity and stability of the composite derived from the physical mixing of Zeo-FPT under investigation were demonstrated by the fact that all the experimental data reported are based on the average of three replicates and had an error of less than 5%. The percentage removal (%) and the adsorption capacity (*Q*) of MB dye retention on the Zeo-FPT sorbent from the aqueous solution were calculated by employing equations that have been applied in previous publications [30].

### 3.5. Sorption Kinetic Study

In the treatment of aqueous effluents, adsorption kinetic studies are crucial because they provide useful information about the adsorption process mechanism. Many kinetic models have been developed to determine fundamental kinetic adsorption constants. The pseudo-first- and second-order models are the most used kinetic models for describing the adsorption process based on chemical reaction kinetics. In this study, the Zeo-FPT had the capacity to adsorb MB dye and was soluble in water [42].

#### 3.5.1. The Pseudo-First-Order Model

The linear form of the pseudo-first-order model is given by Equation (1):

(1) $$\log\left(Q_{e}-Q_{t}\right)=\log Q_{e}-\frac{k_{1}}{2.303}t$$

where *Q_e_* is the adsorption capacity at equilibrium (mg g^−1^), *Q_t_* is the amount of adsorbate adsorbed at time *t* (mg g^−1^), *k*_1_ is the pseudo-first-order rate constant (min^−1^), and *t* is the contact time (min). The values of the adsorption rate constant *k*_1_, equilibrium adsorption capacity, and the correlation coefficient R^2^ were determined from the plot of log *(Q_e_ − Q_t_)* versus *t* at different concentrations.

#### 3.5.2. The Pseudo-Second-Order Model

The linearized form of the second-order kinetic model to calculate the *k*_2_ where the pseudo-second-order rate constant (g mg^−1^ min^−1^) is from the given Equation (2):

(2) $$\frac{t}{Q_{t}}=\frac{1}{k_{2}Q_{e}^{2}}+\frac{1}{Q_{e}}t$$

### 3.6. Adsorption Isotherm Model

The adsorption isotherm, at a constant temperature, provides useful data on the mechanism and surface properties of the adsorbate to adsorbed by the adsorbent and that remaining in solution after reaching equilibrium. Several equations can be used to describe the adsorbate’s adsorption equilibrium on an adsorbent; the most well known are the Langmuir and Freundlich isotherm models [43].

#### 3.6.1. Langmuir Isotherm Model

The Langmuir isotherm model is generally useful under the following conditions:

- An adsorbate monolayer is created on the adsorbent surface when it becomes saturated;
- Adsorbates are adsorbed at a specified number of sites;
- All sites are energetically equivalent;
- All sites hold one adsorbate species;
- No interactions occur between the species of the adsorbate.

The Langmuir model is given by Equation (3):

(3) $$\frac{C_{e}}{Q_{e}}=\frac{1}{K_{l}Q_{m}}+\left(\frac{1}{Q_{m}}\right)C_{e}$$

In this equation, *C_e_* represents the supernatant concentration of the adsorbate (mg L^−1^). *K_l_* represents the equilibrium Langmuir constant associated with the affinity of the binding sites (L mg^−1^), and *Q_max_* represents the maximum capacity of adsorption (mg g^−1^). While applying the linearized Langmuir equation by plotting *C_e_/Q_e_* against *C_e_*, *Q_max_* and *K_l_* were obtained from the slope and the intercept, respectively.

#### 3.6.2. Freundlich Isotherm Model

The Freundlich isotherm model presents an exponential form which assumes that an adsorbate concentration on the surface of the adsorbent increases as the concentration of the adsorbate increases. Therefore, an infinite amount of adsorption can take place when using this model. The model also states that adsorption can occur through several heterogeneous layers. The Freundlich isotherm model is expressed by Equation (4):

(4) $$\log Q_{e}=\left(\frac{1}{n}\right)\log C_{e}+\log K_{F}$$

where *K_F_* represents the constant of the Freundlich equilibrium (mg g^−1^(mg L^−1^)^−1/n^) while 1/*n* represents the Freundlich indicator (dimensionless), where sorption is irreversible if 1/*n* = 0, sorption is unfavorable if 1/*n* > 1, and sorption is favorable if (0 < 1/*n* < 1) [34].

#### 3.6.3. Temkin Isotherm Model

The Temkin model presumes that the adsorbate and adsorbent interaction causes the heat of the adsorption of analytes in the surface layer to decrease linearly with increasing surface coverage. The Timken isotherm model can be applied via Equations (5) and (6):

(5) $$Q_{e}=\frac{RT}{b}LnK_{T}+\frac{RT}{b}\ln C_{e}$$

(6) $$B=\frac{RT}{b}$$

Where *K_T_* corresponds to the Temkin isotherm constant (L g^−1^), R is the gas constant, and *b* is the Temkin constant given by plotting *Q_e_* vs. *ln C_e_* attained from the slope and *K_T_* from the intercept. *B* is a constant associated with the heat of analyte retention (kJ mol^−1^).

### 3.7. Study of Environmental Samples

Environmental water samples (tap water, bottled water, and well water) were filtered to remove impurities. A spiked solution was prepared with 10 mL of 10 mg L^−1^ MB dye solution added to 90 mL of each of the three water samples (tap water, well water, and bottled water). The adsorption was studied for optimum parameters with 0.2 g of (1F:1Z) Zeo-FPT added to 25 mL of the spiked 10 mg L^−1^ MB dye solution at pH 8, with a reaction time of 70 min at room temperature. The samples were filtered using No. 2 Whatman filter paper and analyzed using a UV–vis spectrophotometer.

## 4. Conclusions

In conclusion, fiber of palm tree (FPT) and Saudi kaolin waste were successfully used as starting materials for the preparation of effective solid composites of zeolite and FPT (Zeo-FPT) in a diverse ratio. The newly created (Zeo-FPT) composite exhibited a remarkable efficiency for the removal of MB dye from aqueous solutions. The synthesized zeolite A was characterized as having a shape of rhombohedral crystal, and the MB dye was adsorbed on the surface of Zeo-FPT. The results show that the percentage removal of MB dye in an equal (1F:1Z) ratio of Zeo-FPT was 84.99%, and the removal capacity was 0.438 mg g^−1^ after 40 min at pH 8, with a sorbent mass of 0.5 g and a concentration of 10 mg L^−1^, which are the optimal parameters. The results demonstrate that the kinetics models adsorption mechanism was described well by a pseudo-second-order model with R^2^ 0.998, and the obtained value of the sorption capacity (*Q_e,calc_* 0.353 mg g^−1^) was in agreement with the experimental capacity (*Q_e,exp_* 0.438 mg g^−1^). The adsorption isotherm strongly agreed with that of the Freundlich model, while the R^2^ was 0.969, verifying that the adsorption process was heterogeneous, with multiple layers adsorbed onto the surface of the Zeo-FPT. The data of the environmental water samples indicate that Zeo-FPT has great potential utility as an adsorbent for removing MB dye. We conclude that the combination of low cost and easy synthesis using natural raw materials makes Zeo-FPT an attractive adsorbent—as well as being environmentally friendly and cost-effective—for the removal of MB dyes from wastewater. This study suggests encourages further investigations into the possibility of using a low-cost sorbent, such as kaolin, and fiber of palm tree in the preparation of sorbents, and enhances the economic utility of these prepared materials.

## Figures and Tables

**Table 1:** Lattice constant of zeolite A.

Sample	a	b	c	α	β	γ	Formula
Zeolite A, reference	12.144	12.144	12.144	90	90	90	(Li, Na)_2_ Al_2_ Si_1.85_ O_7.7_ × H_2_

**Table 2:** FTIR spectroscopy absorption bands of Zeo, FPT, and the Zeo-FPT composite.

Wave Number cm^−1^	Stretching	Bending
518–478	stretching of Zeo-FPT vibrations of the bonds inside the TO_4_ (T = Al, Si) tetrahedrons	weak bands of Zeo-FPT bending vibrations of O–T–O
670–650	weak bands of symmetric stretching vibration of the T–O bonds (Si–O–Si bending)	
1000–952	Zeo and Zeo-FPT, the internal asymmetric stretching vibration of Si–O–T (T = Si or Al)	C=C bending; strong monosubstituted alkene
1000	strong, broad CO–O–CO stretching anhydride	
1044	the internal asymmetric stretching vibration of Si–O–T (T = Si or Al) in zeolite	
1500		bending vibration bands of strong hydroxyl groups
2165–2030	triple bond region (2000–2500) presence of alkynes C=C=N stretching ketenimine C=C=O stretching ketene	
3200–2700 cm^−1^	weak broad O–H stretching	weak bending vibration bands of hydroxyl groups (H–O–H)
3740	stretching vibrations of the OH groups; vibration found for water vapor	

**Table 3:** Adsorption kinetics parameters calculated for the pseudo-first- and pseudo-second-order kinetics for the removal of MB using Zeo-FPT.

Pseudo-First-Order		Pseudo-Second-Order	
*Q_e,calc_* (mg g^−1^)	0.220	*Q_e,calc_* (mg g^−1^) *Q_e,exp_* (mg g^−1^)	0.353 0.438
R^2^	0.748	R^2^	0.998
*K*_1_ (min^−1^)	0.016	*K*_2_ (g mg^−1^ min^−1^)	0.787

**Table 4:** Adsorption isotherm parameters calculated for the removal of MB using Zeo-FPT.

Isotherm Model	Parameter	Value
Langmuir	*Q_max_* (mg g^−1^)	27.933
	*K_L_* (L mg^−1^)	4.533 × 10^−3^
	R^2^	0.547
Freundlich	*K_f_* (mg^1−1/n^ L^1/n^ g^−1^)	0.163
	1/n	0.884
	R^2^	0.969
Temkin	*K_T_* (L g^−1^)	1.57
	R^2^	0.732
	*B* (kJ mol^−1^)	9.36

**Table 5:** A comparison between the sorption characteristics of the established Zeo-FPT (1:1) biosorbent and some other biosorbents used for MB dye removal.

Biosorbent	*Q_max_* mgg^−1^	Adsorption Time (min)	Kinetic/Isotherm Models	Reference
Zeolite modified by magnetic graphene oxide (Cu-Z-GO-M) (1:1)	99.2	600	(PSO)/(FRH)	[38]
Sugarcane waste-activated carbon modified with natural zeolite (AC500/NZ)	51	-	(PSO)/(TMK)	[21]
Zeolite 4A from Ethiopian kaolin	44.35	179.82	(PSO)/(LNR)	[13]
Zeolite-X from Ethiopian kaolin	0.61	180	(PSO)/(FRH)	[6]
Clove leaves activated with sodium hydroxide (CL-NaOH)	9.80	60	(PSO)/(LNR)	[39]
Rice straw biochars	131.58	-	(PSO)/(LNR)	[40]
(Zeo-FPT) (1:1) biosorbent	0.438	40	(PSO)/(FRH)	this study

Freundlich (FRH), Langmuir (LNR), Temkin (TMK), pseudo-first-order (PFO) and pseudo-second-order (PSO) models.

**Table 6:** The removal percentage and adsorption capacity at fixed parameters for environmental samples.

Water	Time	m (g)	C (mg L^−1^)	V (L)	%	*Q* (mg g^−1^)
	min					
Tap	70	0.2	10	0.025	99.040	1.238
Well	70	0.2	10	0.025	99.382	1.242
Bottle	70	0.2	10	0.025	97.856	1.223

## Data Availability

Not applicable.

## Sample Availability

Samples of the compound are not available from the authors.

## References

[1] Hassan M.M., Carr C.M. (2021). Biomass-derived porous carbonaceous materials and their composites as adsorbents for cationic and anionic dyes: A review. Chemosphere.

[2] Tani A., Thomson A.J., Butt J.N. (2001). Methylene blue as an electrochemical discriminator of single- and double-stranded oligonucleotides immobilised on gold substrates. Analyst.

[3] Bożęcka A.M., Orlof-Naturalna M.M., Kopeć M. (2021). Methods of Dyes Removal from Aqueous Environment. J. Ecol. Eng..

[4] Allafchian A., Mousavi Z.S., Hosseini S.S. (2019). Application of cress seed musilage magnetic nanocomposites for removal of methylene blue dye from water. Int. J. Biol. Macromol..

[5] Hassan A., Abdel-Mohsen A., Fouda M.M. (2014). Comparative study of calcium alginate, activated carbon, and their composite beads on methylene blue adsorption. Carbohydr. Polym..

[6] Mulushewa Z., Dinbore W.T., Ayele Y. (2021). Removal of methylene blue from textile waste water using kaolin and zeolite-x synthesized from Ethiopian kaolin. Environ. Anal. Health Toxicol..

[7] Quan X., Luo D., Wu J., Li R., Cheng W., Ge S. (2017). Ozonation of acid red 18 wastewater using O_3_/Ca(OH)_2_ system in a micro bubble gas-liquid reactor. J. Environ. Chem. Eng..

[8] Musz-Pomorska A., Widomski M.K. (2020). Variant analysis of the chlorination efficiency of water in a selected water supply network. Proc. ECOpole’19 Conf..

[9] Tahir K., Miran W., Jang J., Maile N., Shahzad A., Moztahida M., Ghani A.A., Kim B., Jeon H., Lee D.S. (2021). MXene-coated biochar as potential biocathode for improved microbial electrosynthesis system. Sci. Total Environ..

[10] Biedrzycka A., Skwarek E., Hanna U.M. (2021). Hydroxyapatite with magnetic core: Synthesis methods, properties, adsorption and medical applications. Adv. Colloid Interface Sci..

[11] Babu J., Murthy Z. (2017). Treatment of textile dyes containing wastewaters with PES/PVA thin film composite nanofiltration membranes. Sep. Purif. Technol..

[12] Sarayu K., Sandhya S. (2012). Current Technologies for Biological Treatment of Textile Wastewater—A Review. Appl. Biochem. Biotechnol..

[13] Belachew N., Hinsene H. (2022). Preparation of Zeolite 4A for Adsorptive Removal of Methylene Blue: Optimization, Kinetics, Isotherm, and Mechanism Study. Silicon.

[14] Neolaka Y.A., Riwu A.A., Aigbe U.O., Ukhurebor K.E., Onyancha R.B., Darmokoesoemo H., Kusuma H.S. (2023). Potential of activated carbon from various sources as a low-cost adsorbent to remove heavy metals and synthetic dyes. Results Chem..

[15] Xue H., Gao X., Seliem M.K., Mobarak M., Dong R., Wang X., Fu K., Li Q., Li Z. (2023). Efficient adsorption of anionic azo dyes on porous heterostructured MXene/biomass activated carbon composites: Experiments, characterization, and theoretical analysis via advanced statistical physics models. Chem. Eng. J..

[16] Zhou Y., Lu J., Zhou Y., Liu Y. (2019). Recent advances for dyes removal using novel adsorbents: A review. Environ. Pollut..

[17] El Bardiji N., Ziat K., Naji A., Saidi M. (2020). Fractal-Like Kinetics of Adsorption Applied to the Solid/Solution Interface. ACS Omega.

[18] Łach M., Grela A., Pławecka K., Guigou M.D., Mikuła J., Komar N., Bajda T., Korniejenko K. (2022). Surface Modification of Synthetic Zeolites with Ca and HDTMA Compounds with Determination of Their Phytoavailability and Comparison of CEC and AEC Parameters. Materials.

[19] Neolaka Y.A., Lawa Y., Riwu M., Darmokoesoemo H., Setyawati H., Naat J., Widyaningrum B.A., Aigbe U.O., Ukhurebor K.E., Onyancha R.B. (2022). Synthesis of Zinc(II)-natural zeolite mordenite type as a drug carrier for ibuprofen: Drug release kinetic modeling and cytotoxicity study. Results Chem..

[20] Dosa M., Grifasi N., Galletti C., Fino D., Piumetti M. (2022). Natural Zeolite Clinoptilolite Application in Wastewater Treatment: Methylene Blue, Zinc and Cadmium Abatement Tests and Kinetic Studies. Materials.

[21] Mohamed F., Shaban M., Zaki S.K., Abd-Elsamie M.S., Sayed R., Zayed M., Khalid N., Saad S., Omar S., Ahmed A.M. (2022). Activated carbon derived from sugarcane and modified with natural zeolite for efficient adsorption of methylene blue dye: Experimentally and theoretically approaches. Sci. Rep..

[22] Van Dang L., Nguyen T.T.M., Van Do D., Le S.T., Pham T.D., Le A.T.M. (2021). Study on the Synthesis of Chabazite Zeolites via Interzeolite Conversion of Faujasites. J. Anal. Methods Chem..

[23] Kadja G.T., Culsum N.T., Mardiana S., Azhari N.J., Fajar A.T. (2022). Recent advances in the enhanced sensing performance of zeolite-based materials. Mater. Today Commun..

[24] Allbed A., Kumar L., Shabani F. (2017). Climate change impacts on date palm cultivation in Saudi Arabia. J. Agric. Sci..

[25] Chowdhury S., Pan S., Balasubramanian R., Das P. (2019). Date palm based activated carbon for the efficient removal of organic dyes from aqueous environment. Sustain. Agric. Rev..

[26] Elseify L.A., Midani M., Shihata L.A., El-Mously H. (2019). Review on cellulosic fibers extracted from date palms (*Phoenix dactylifera* L.) and their applications. Cellulose.

[27] Domingues J.A., Consolin Filho N., Souza LA G.D., Medeiros F.V.D.S. (2020). Coagulation activity of the seed extract from *Zygia cauliflora* (WILLD.) KILLIP Applied in Water Treatment. Rev. Ambiente Água.

[28] Shafiq M., Alazba A.A., Amin M.T. (2018). Removal of heavy metals from wastewater using date palm as a biosorbent: A comparative review. Sains Malays..

[29] Asim M., Jawaid M., Khan A., Asiri A.M., Malik M.A. (2020). Effects of Date Palm fibres loading on mechanical, and thermal properties of Date Palm reinforced phenolic composites. J. Mater. Res. Technol..

[30] Alhogbi B.G., Altayeb S., Bahaidarah E.A., Zawrah M.F. (2021). Removal of Anionic and Cationic Dyes from Wastewater Using Activated Carbon from Fiber of palm tree Waste. Processes.

[31] El Maksod I.A., Elzaharany E.A., Kosa S.A., Hegazy E.Z. (2016). Simulation program for zeolite A and X with an active carbon composite as an effective adsorbent for organic and inorganic pollutants. Microporous Mesoporous Mater..

[32] Neto J.S.S., de Queiroz H.F.M., Aguiar R.A.A., Banea M.D. (2021). A Review on the Thermal Characterisation of Natural and Hybrid Fiber Composites. Polymers.

[33] Mosai A.K., Chimuka L., Cukrowska E.M., Kotzé I.A., Tutu H. (2019). The Recovery of Rare Earth Elements (REEs) from Aqueous Solutions Using Natural Zeolite and Bentonite. Water Air Soil Pollut..

[34] Dhiman V., Kondal N. (2021). ZnO nanoadsorbents: A potent material for removal of heavy metal ions from wastewater. Colloid Interface Sci. Commun..

[35] Dehghani M.H., Dehghan A., Alidadi H., Dolatabadi M., Mehrabpour M., Converti A. (2017). Removal of methylene blue dye from aqueous solutions by a new chitosan/zeolite composite from shrimp waste: Kinetic and equilibrium study. Korean J. Chem. Eng..

[36] Gao W., Zhao S., Wu H., Deligeer W., Asuha S. (2016). Direct acid activation of kaolinite and its effects on the adsorption of methylene blue. Appl. Clay Sci..

[37] Li C., Zhong H., Wang S., Xue J., Zhang Z. (2015). Removal of basic dye (methylene blue) from aqueous solution using zeolite synthesized from electrolytic manganese residue. J. Ind. Eng. Chem..

[38] Huang T., Yan M., He K., Huang Z., Zeng G., Chen A., Peng M., Li H., Yuan L., Chen G. (2019). Efficient removal of methylene blue from aqueous solutions using magnetic graphene oxide modified zeolite. J. Colloid Interface Sci..

[39] Kusuma H.S., Aigbe U.O., Ukhurebor K.E., Onyancha R.B., Okundaye B., Simbi I., Ama O.M., Darmokoesoemo H., Widyaningrum B.A., Osibote O.A. (2023). Biosorption of Methylene blue using clove leaves waste modified with sodium hydroxide. Results Chem..

[40] Wang K., Peng N., Sun J., Lu G., Chen M., Deng F., Dou R., Nie L., Zhong Y. (2020). Synthesis of silica-composited biochars from alkali-fused fly ash and agricultural wastes for enhanced adsorption of methylene blue. Sci. Total Environ..

[41] Ibrahim H., Jamil T.S., Hegazy E. (2010). Application of zeolite prepared from Egyptian kaolin for the removal of heavy metals: II. Isotherm models. J. Hazard. Mater..

[42] Sandollah N.A.S., Ghazali S.A.I.S.M., Ibrahim W.N.W., Rusmin R. (2020). Adsorption-Desorption Profile of Methylene Blue Dye on Raw and Acid Activated Kaolinite. Indones. J. Chem..

[43] Ruthven D.M. (1984). Principles of Adsorption and Adsorption Processes.

